# Contraceptive Use Before and After Abortion: A Cross‐Sectional Study from Nigeria and Côte d'Ivoire

**DOI:** 10.1111/sifp.12208

**Published:** 2022-07-20

**Authors:** Sophia Magalona, Meagan Byrne, Funmilola M. OlaOlorun, Rosine Mosso, Elizabeth Omoluabi, Caroline Moreau, Suzanne O. Bell

**Affiliations:** ^1^ Sophia Magalona is a PhD student, Meagan Byrne is PMA Survey Operations Lead, Caroline Moreau is Associate Professor, and Suzanne O. Bell is Assistant Professor, Department of Population Family and Reproductive Health Johns Hopkins Bloomberg School of Public Health Baltimore Maryland USA; ^2^ Funmilola M. OlaOlorun is Senior Lecturer, Department of Community Medicine University of Ibadan Ibadan Oyo Nigeria; ^3^ Rosine Mosso is Lecturer and Researcher École Nationale Supérieure de Statistique et d'Économie Appliquée Abidjan Côte d'Ivoire; ^4^ Elizabeth Omoluabi is Managing Director, Centre for Research Evaluation Resources and Development Ile‐Ife Nigeria; ^5^ Caroline Moreau is Epidemiologist, CESP Centre for Research in Epidemiology and Population Health INSERM (Institut National de la Santé et de la Recherche Médicale) Villejuif France

**Keywords:** abortion, contraception, family planning

## Abstract

Post‐abortion contraception enables women to effectively manage their fertility to prevent unintended pregnancies. Using data from population‐based surveys of women aged 15–49 in Nigeria and Côte d'Ivoire, we examined contraceptive dynamics immediately before and after an abortion and examined factors associated with these changes using multivariable logistic regressions. Covariates included sociodemographic characteristics, abortion source, post‐abortion contraceptive communication (wanting to and actually talking to someone about contraception after abortion), and perceived contraceptive autonomy. We observed higher contraceptive use after abortion than before abortion. In Nigeria, wanting to talk to someone about contraception post‐abortion was associated with increased adoption and decreased discontinuation, whereas talking to someone about contraception post‐abortion was associated with increased adoption. Obtaining care from a clinical abortion source was associated with increased adoption and decreased discontinuation. Both post‐abortion contraceptive communication variables were associated with post‐abortion contraceptive use in both countries, whereas clinical source was only associated with post‐abortion contraceptive use in Nigeria. Our findings suggest that ensuring that women have access to safe abortion as part of the formal health care system and receive comprehensive, high‐quality post‐abortion care services that include contraceptive counseling enables them to make informed decisions about their fertility that align with their reproductive goals.

## BACKGROUND

Abortion is a safe health care intervention when conducted in accordance with recommended guidelines, yet unsafe abortion remains prevalent and a leading cause of maternal mortality, especially in settings where it is legally restricted (Say et al. 2014; Ganatra et al. 2017). Globally, 45 percent of abortions are considered unsafe (Ganatra et al. 2017). In sub‐Saharan Africa, most women of reproductive age (WRA; 15–49 years old) live in countries that prohibit abortion or only allow it to save a woman's life. Out of 8 million unintended pregnancies that ended in abortions every year between 2015 and 2019 in the region, 77 percent were considered unsafe, leading to 185 maternal deaths per 100,000 abortions—the highest of any world region (Bankole et al. 2020). Contraception enables women to effectively manage their fertility to prevent unintended pregnancies and potential complications arising from unsafe abortions in settings where access to safe abortion care is restricted. For many women, their abortion experience presents an opportunity to receive voluntary contraceptive counseling and methods (High Impact Practices in Family Planning 2019; Curtis et al. 2010) at a time when they may be more motivated to use a method and may already be in contact with a health care provider via their abortion or post‐abortion care (PAC).

PAC services have been a component of the health system since the 1990s, when countries pledged to provide quality interventions to prevent unsafe abortion‐related morbidity and mortality (Tripney et al. 2013; Corbett and Turner 2003). One of the key elements of PAC is family planning counseling and services, making a range of contraceptive options and information available to women to help them fulfill their reproductive goals to space or limit births. Providing this service at the same visit and place as other PAC services is ideal, as fertility returns in as few as eight days after an abortion and women are already in contact with a health facility (Boyd and Holmstrom 1972; Barot 2014). However, despite the safety and efficacy of post‐abortion contraception, service availability varies widely across settings. A multicountry analysis of PAC provision found that while most countries include contraceptive counseling in PAC policies and national guidelines, service provision ranged from 0 percent to 29 percent of primary‐level facilities across settings (Owolabi et al. 2019). In addition, women continue to face challenges with accessing care, such as high costs or stigma reinforced by legal restrictions that may make them reluctant to openly seek PAC services (Corbett and Turner 2003; Barot 2014; Shearer et al. 2010). The increasing availability of medication abortion outside of the formal health care system to induce abortion overcomes some of these barriers as a safe, effective, and inexpensive option that allows for self‐administration (Barot 2014); however, information on where women go to access medication abortion and whether they receive adequate information for proper use is limited (Bankole et al. 2020). Although medication abortion is available in clinical settings, clients may also access them via pharmacies and drug shops, which are often preferred options for health care sources in low‐resource settings due to convenience, privacy, and low cost. However, the quality of medication abortion provision is often poor, with clients not receiving adequate, or sometimes any, information (Footman et al. 2018). Furthermore, contraceptive counseling is typically not part of the care experience in these circumstances.

Studies that have looked at contraceptive behavior before and after an abortion show improved contraceptive uptake (Baynes et al. 2019; Moseson et al. 2018; Benson et al. 2018; Hagos et al. 2018; Makenzius et al. 2018; Macha et al. 2018) and adoption of more effective methods post‐abortion compared to pre‐abortion (Moseson et al. 2018; Moreau et al. 2010, 2012; Madden et al. 2011). An analysis using public hospital data from Ethiopia, Ghana, Nigeria, South Africa, and Zambia observed contraceptive uptake levels ranging from 42 percent in South Africa to 86 percent in Ethiopia, with most women opting for injectables (Benson et al. 2018). The literature that examined factors associated with post‐abortion contraception found that women who received family planning counseling and services were more likely to use contraception than their counterparts (Hagos et al. 2018; Moges et al. 2018; Tripney et al. 2013). In addition, provider type or provider training was found to be associated with contraceptive uptake (Benson et al. 2017; Maxwell et al. 2015). A multicountry evaluation of an Ipas program that provided post‐abortion contraceptive support in health facilities found that women who interacted with a provider who received Ipas training were more likely to use contraception post‐abortion (Benson et al. 2017). Facility type was also a factor of interest in some studies, but the results were less clear, with some analyses finding women more likely to use contraception when they access abortion care services at public facilities compared to private clinics (Hagos et al. 2018; Moges et al. 2018), whereas others did not observe this association (Benson et al. 2017; Prata et al. 2011). Other variables associated with post‐abortion contraceptive uptake include marital status, education level, previous contraceptive use, and parity (Hagos et al. 2018; Moges et al. 2018; Prata et al. 2011).

Despite findings that provide insight into post‐abortion contraceptive use dynamics, the existing literature has several limitations. First, studies are often limited in the factors that they can explore because data from low‐ and middle‐income countries (LMICs) mainly use facility‐based samples that rely on facility records or client logbooks. These data sources only include a few patient characteristics and whether the patient received PAC but do not capture information about the quality of the interaction, who the patient interacted with, fertility intentions or contraceptive preferences (Baynes et al. 2019; Benson et al. 2018; Macha et al. 2018; Maxwell et al. 2015). Second, these data lack information on contraceptive use prior to abortion, limiting the outcome of analyses to post‐abortion contraceptive use only. Finally, much of the existing literature on post‐abortion contraceptive dynamics from LMICs draws from public and private facilities only (Baynes et al. 2019; Benson et al. 2018; Hagos et al. 2018; Macha et al. 2018; Makenzius et al. 2018), excluding women who access sources outside of the formal health care system, such as pharmacies and drug shops, where medical abortion is widely available (World Health Organization 2012).

To fill these three gaps, our study used population‐based samples of WRA who reported an abortion—including those terminated entirely outside the formal health care system—in two West African countries, Nigeria and Côte d'Ivoire, which were part of a larger, multicountry study on abortion incidence and safety. In Nigeria, induced abortion is legal only if performed to save a woman's life (though more legal indications exist in some Nigerian states), whereas in Côte d'Ivoire, it is legal to save a woman's life and in cases of rape or incest. Despite these restrictions, abortions are a common means by which women manage their fertility. Recent studies from the Performance Monitoring for Action (PMA) project estimated a one‐year abortion rate of 45.8 per 1,000 WRA in Nigeria and 40.7 per 1,000 WRA in Côte d'Ivoire in 2017; these estimates are higher than the 32 abortions per 1,000 WRA annual estimate for the West Africa region between 2015 and 2019 (Bankole et al. 2020; Bell et al. 2020a, 2020c). In the same PMA studies, approximately two‐thirds of abortions in both countries were categorized as most unsafe (63.4 percent in Nigeria and 62.4 percent in Côte d'Ivoire), defined as involving nonrecommended methods (procedures other than surgery or medication abortion drugs) and nonclinical or no providers (Bell et al. 2020a, 2020c). These are higher than global estimates, which suggest that 52 percent of abortions in West Africa are unsafe (Bankole et al. 2020). Additional research similarly suggests that nonclinical sources are common in both countries. A traditional provider or “other” source, which includes markets, friends or relatives, or home, was the most frequent source used to terminate a pregnancy in Nigeria (50.2 percent) and Côte d'Ivoire (56.8 percent) (Bell et al. 2019). A separate study in Nigeria found that approximately one‐third of women sought services from nonclinical sources to terminate their pregnancy before presenting for care at a hospital (Henshaw et al. 2008). Nonclinical sources often lack appropriate training and typically induce bleeding by providing herbs, tablets, or insert objects into women's bodies—procedures that are often not only ineffective but also life‐threatening (Bankole et al. 2006). Following these unsafe terminations, many women find themselves in need of PAC; however, a study assessing facility readiness for PAC provision in both countries found that less than half (48.4 percent) of health facilities in Nigeria provided basic services, which include contraceptive availability, whereas in Côte d'Ivoire, this percentage was higher (70.5 percent) (Bell et al. 2021).

Both countries have among the lowest levels of contraceptive use in the region, with only 24 percent and 29 percent of WRA using any method in Nigeria in 2018 and Côte d'Ivoire in 2020, respectively. However, both countries show a gradual increase in contraceptive use in recent years, from 20 percent in 2016 to 24 percent in 2018 in Nigeria and 26 percent in 2017 to 29 percent in 2020 in Côte d'Ivoire (Performance Monitoring for Action 2018, 2021). Understanding contraceptive dynamics and factors associated with contraceptive uptake after an abortion is crucial in these settings, where many unintended pregnancies are terminated unsafely (contributing to high levels of maternal mortality) and contraceptive prevalence remains low.

The overall goal of the study is to examine changes in contraceptive behavior among women who terminated a pregnancy in Nigeria and Côte d'Ivoire. Specifically, we aim to (1) describe contraceptive dynamics before and after abortion, (2) examine factors associated with contraceptive adoption, discontinuation, and switching to more effective methods after abortion, and (3) assess correlates of contraceptive use after an abortion.

## METHODS

### Study Overview

This study used data from the PMA Project. PMA collects rapid and frequent national or regional population‐based survey data in nine countries in Africa and Asia. The project employs resident enumerators—local women—who collect information in their communities on women's birth history, fertility preferences, and contraceptive practices using smartphones to enter data during face‐to‐face interviews. Full details of the PMA project, including the study and sampling design, questionnaires, and data, are available from www.pmadata.org and Zimmerman et al. (2017). For this study, we used data from Nigeria Round 5 and Côte d'Ivoire Round 2 and associated follow‐up surveys. Implementing partners were the Centre for Research, Evaluation Resources and Development in Nigeria and the Institut National de la Statistique, the Coordination du Programme National de Santé de la Mère et de l'Enfant within the Ministry of Health, and the École Nationale Supérieure de Statistique et d'Économie Appliquée (ENSEA) in Côte d'Ivoire. The Bill & Melinda Gates Institute at the Johns Hopkins Bloomberg School of Public Health provided technical support and overall project direction.

The sampling strategies at baseline slightly differed between the two countries, but both generated nationally representative samples of WRA. In Nigeria, a three‐stage cluster sampling approach was used. The first stage consisted of selecting seven states using probability proportional to size (PPS) sampling, followed by the selection of geographic areas within each state also using PPS sampling. In Côte d'Ivoire, a two‐stage cluster sampling design with urban–rural stratification was used to select geographic clusters. The last stage of sampling was the same for both countries. Each cluster represented approximately 200 households in both settings. All households within the selected cluster were mapped and listed, and 35 (40 in Lagos state, Nigeria) households within each cluster were randomly selected and invited to participate. Heads from selected households completed the household interview. All women aged 15–49 identified in the selected households were eligible to participate in the women's face‐to‐face interviews. Interviews were conducted primarily in French in Côte d'Ivoire and Hausa, Igbo, Pidgin, Yoruba, and English in Nigeria. In Côte d'Ivoire, interviewers also used agreed upon oral translations to conduct interviews in local dialects, as needed.

Baseline data collection occurred from April to May 2018 in Nigeria and from July through August 2018 in Côte d'Ivoire. At baseline, respondents were asked about possible abortions using different terminology instead of asking directly about abortion because of the stigmatization of the event and to improve the quality of self‐reporting. Women were asked separately whether they had done something to “remove a pregnancy” or to bring back a late period at a time when the respondent thought she was pregnant (hereafter referred to as period regulation). Language for period regulation was included to capture the experiences of women who may have nuanced interpretations of a possible pregnancy termination, especially when their pregnancy status is ambiguous or has not been confirmed clinically or via pregnancy test (Bell and Fissell 2021; Sheehy et al. 2021; Bell et al. 2020b). The phrasings used to capture pregnancy removal or period regulation experiences emerged from pilot training in Nigeria during discussions with female data collectors about the language women use to discuss actions that could be classified as abortion. In both countries, the questionnaires were updated with these specific phrasings and pilot tested prior to data collection to confirm the comprehension and interpretation of both interviewers and respondents. Interviewers and respondents interpreted the phrasings of these different abortion experiences correctly (Bell et al. 2020b). In this analysis, experiences of removing a pregnancy and regulating a period were both classified as abortions.

Respondents who reported an abortion during the baseline survey and consented to be recontacted to discuss their abortion experience were contacted to participate in a follow‐up survey. The follow‐up surveys were conducted from December 2019 to February 2020 in Nigeria and October to November 2020 in Côte d'Ivoire. The follow‐up survey asked about the respondent's abortion experience using the language previously reported by the respondent at baseline (pregnancy removal or period regulation). All participants provided verbal informed consent prior to both the baseline and follow‐up survey. Ethical approval was obtained from the Johns Hopkins Bloomberg School of Public Health Institutional Review Board, the National Health Research Ethics Committee of Nigeria, and the Comité National d'Ethique des Sciences de la Vie et de la Santé (CNESVS) of the Côte d'Ivoire Ministry of Health and Public Hygiene.

### Analytical Sample

**FIGURE 1:**
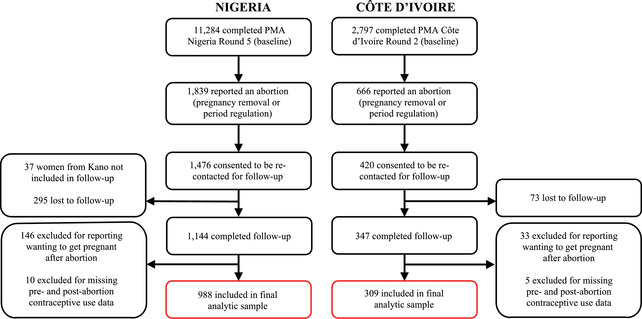
Flowchart of the analytic sample for Nigeria and Côte d'Ivoire

The analytic sample for this study relied on the sample of women who completed the follow‐up survey (Figure 1). Out of 1,476 and 420 women who completed the baseline survey, reported an abortion, and consented to be recontacted in Nigeria and Côte d'Ivoire, respectively, 1,144 (79.5 percent) and 347 (82.6 percent) completed the follow‐up interviews in Nigeria and Côte d'Ivoire, respectively. Women who reported no contraceptive use after abortion were asked “Tell me which of the following describes your situation after the pregnancy ended,” to which one of the answer options was “wanted to become pregnant.” We excluded women who reported wanting to become pregnant after their abortion and further restricted the sample to only women who had data on contraceptive use before and after their abortion, yielding final analytical samples of 988 in Nigeria and 309 in Côte d'Ivoire.

In Nigeria, very few women (n = 37) from Kano reported an abortion at baseline and consented to be recontacted. We excluded them from the follow‐up survey for logistical reasons. Compared to our final analytical sample, the women from Kano were more likely to be older, have higher parity, have never gone to school, are currently married or cohabitating, are in middle or lower wealth tertiles, and reside in rural areas (data not shown).

### Measures

Respondents were asked “Immediately before this event, were you or your partner using anything to avoid or delay getting pregnant?” for pre‐abortion contraceptive use and “After this event, did you begin using contraception to avoid another pregnancy?” for post‐abortion contraceptive use. If they responded “yes,” they were then asked to specify the contraceptive method. Responses to these two questions were used to generate the three outcomes for our first aim to describe contraceptive dynamics before and after an abortion. For our first outcome, we categorized respondents by contraceptive use status, including specific details regarding method type, resulting in the following three categories: no method, short‐acting method, or long‐acting method. Short‐acting methods included pills, injectables, diaphragms, condoms, other barrier methods, traditional methods, emergency contraception, and lactational amenorrhea; long‐acting methods included sterilization, intrauterine devices (IUDs), and implants. We calculated these three categories separately before and after the abortion. For our second outcome, we grouped respondents by change in contraceptive behavior: adopters (changed from nonuser to user), discontinuers (changed from user to nonuser), continued users, or continued nonusers. For our third outcome, we assigned contraceptive users to three groups based on method effectiveness, similar to the study by Karp et al. (2021) and to the categorization by the Center of Disease Control and Prevention (Curtis et al. 2016): highly effective long‐acting (sterilization, IUD, and implant), effective short‐acting (pills, injectables, and diaphragm), and less effective short‐acting (condoms, other barrier methods, traditional methods, emergency contraception, and lactational amenorrhea method). Our second aim was to examine factors associated with contraceptive adoption, discontinuation, and switching to more effective methods post‐abortion separately. For the adoption analysis, our outcome was a binary variable of post‐abortion contraceptive adoption among pre‐abortion contraceptive nonusers (change from nonuser to user). For the discontinuation analysis, our outcome was a binary variable of post‐abortion contraceptive discontinuation among pre‐abortion contraceptive users (change from user to nonuser). For the switching analysis, our outcome was a binary measure of shifting to a more effective contraceptive method after abortion among continued users. Users were assigned the value of 1 if they shifted from less effective short‐acting methods to effective or highly effective methods or from effective to highly effective methods. For our third aim, we used a binary variable of contraceptive use (any method vs. no method) after abortion for our outcome.

Covariates considered related to contraceptive use dynamics included abortion source, abortion method, contraceptive communication and communication preferences, and perceived contraceptive autonomy. Details on the year of abortion, abortion source, abortion method, and household wealth came from the baseline survey, while all other variables were collected in the follow‐up survey. For abortion source, we categorized respondents’ last (if they used more than one source) or only source of abortion into clinical or nonclinical sources. Clinical sources included government hospitals, government health centers, family planning clinics, mobile clinics (public and private), private hospitals, and private doctors, while nonclinical sources included pharmacies, chemists, public events, fieldworkers (private), shops, friends/relatives, healers, markets, and other sources. We categorized abortion method as medical (mifepristone and/or misoprostol), surgical, or other (other pills, injection, traditional methods or other). Contraceptive communication captured desired conversations (wanted or did not want to talk to someone about contraception) and actual conversations (talked or did not talk to someone about contraception) at the time of the abortion. Perceived contraceptive autonomy variables were limited to post‐abortion contraceptive users only. They captured whether respondents felt they had control over their contraceptive use (“Did you feel you had a choice about whether to use contraception?”) and method choice (“Did you feel you had a choice about which contraceptive method to use?”) after an abortion. Sociodemographic characteristics at the time of abortion were also collected at follow‐up, including age, education, marital status, residence and parity. Household wealth was generated using data from the household interview and linked to the women's follow‐up data; thus, it does not capture wealth at the time of the abortion.

### Analytical Methods

For our first aim, we described contraceptive dynamics using the three outcomes: contraceptive use, change in contraceptive behavior, and method effectiveness before and after abortion among continued users. We ran bivariate analyses on changes in contraceptive behavior to assess associated factors. Multivariable logistic regression models were limited to the sample in Nigeria due to the restricted sample size in Côte d'Ivoire. Two separate models examined (1) contraceptive adoption among pre‐abortion contraceptive nonusers and (2) contraceptive discontinuation among pre‐abortion users. A third logistic regression model examined the shift to a more effective contraceptive method after abortion among continued users who were not already using highly effective methods before abortion. For our second aim, we conducted bivariate analyses and multivariable logistic regression for each country using a binary outcome of contraceptive use after abortion with pre‐abortion contraceptive use (no method, short‐acting, or long‐acting) as a covariate.

We checked for collinearity between covariates and set a cutoff of 0.60. Abortion source and type of abortion method were highly correlated (0.69); thus, we only retained abortion source in the analyses. Missingness across covariates did not exceed 1.3 percent; therefore, we performed a complete case analysis. We assessed model fit using the Hosmer–Lemeshow goodness‐of‐fit test.

All analyses account for clustering at the EA level to correctly calculate standard errors. We conducted all analyses in Stata version 16.1 (StataCorp 2019).

## RESULTS

Table 1 shows the distributions of the sample characteristics at the time of abortion for each country. Women in Nigeria were primarily in their 20s (48.6 percent) and mostly married (56.4 percent), whereas a larger proportion (45.1 percent) of women in Côte d'Ivoire was less than 20 years old, and only 44.0 percent were married. In both settings, a higher proportion of women had children at the time of abortion (54.3 percent in Nigeria and 57.6 percent in Côte d'Ivoire). A majority of women in Nigeria attended secondary or higher education (78.7 percent), and 43.9 percent were in the highest wealth tertile, while almost a third (32.0 percent) of women in Côte d'Ivoire had never attended school, and only 32.1 percent were in the highest wealth tertile. Most women in both samples lived in urban areas (62.9 percent in Nigeria and 61.5 percent in Côte d'Ivoire).

**TABLE 1:** Sociodemographic and contraceptive characteristics of women who reported having an abortion in Nigeria and Côte d'Ivoire

	Nigeria		Côte d'Ivoire	
	n	%	n	%
N	988		309	
Age				
<20	219	22.5	137	45.1
20‐29	474	48.6	121	39.8
30+	282	28.9	46	15.1
Education				
Never	95	9.6	99	32.0
Primary	116	11.8	119	38.5
Secondary (combined with higher for CDI)	505	51.2	91	29.4
Higher	271	27.5	–	
Married				
No	430	43.6	173	56.0
Yes	557	56.4	136	44.0
Wealth				
Lowest	200	20.3	95	30.8
Middle	353	35.8	114	37.0
Highest	432	43.9	99	32.1
Residence				
Rural	367	37.1	119	38.5
Urban	621	62.9	190	61.5
Had a child/children				
No	452	45.7	131	42.4
Yes	536	54.3	178	57.6
Contraceptive use before abortion				
No	572	57.9	209	67.6
Yes	416	42.1	100	32.4
Contraceptive use after abortion				
No	405	41.0	160	51.8
Yes	583	59.0	149	48.2

NOTE: Proportions (%) account for clustering at the EA level.

CDI, Côte d'Ivoire.

### Aim 1: Contraceptive Dynamics Before and After Abortion

**FIGURE 2:**
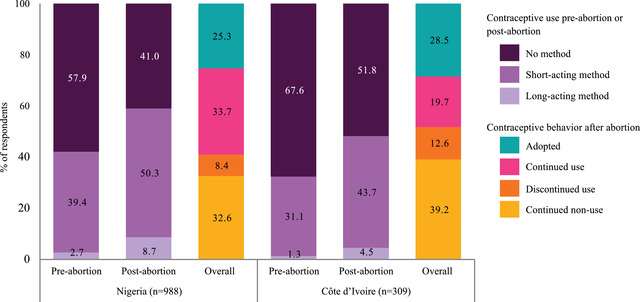
Contraceptive use and behavior before and after abortion in Nigeria and Côte d'Ivoire

Contraceptive use before abortion was relatively low in both settings at 42.1 percent in Nigeria and 32.4 percent in Côte d'Ivoire (Table 1 and Figure 2). The levels of contraceptive use increased following abortion by at least 15 percentage points in both countries to 59.0 percent and 48.2 percent, respectively. Short‐acting methods were much more common (39.4 percent in Nigeria and 31.1 percent in Côte d'Ivoire) than long‐acting methods (2.7 percent in Nigeria and 1.3 percent in Côte d'Ivoire) prior to abortion (Figure 2). Short‐acting method use increased by at least 10 percentage points to 50.3 percent in Nigeria and 43.7 percent in Côte d'Ivoire following abortion. Although the use of long‐acting methods remained low at 8.7 percent in Nigeria and 4.5 percent in Côte d'Ivoire, the relative increases of more than three times the pre‐abortion level are notable.

Figure 2 also shows contraceptive behavior post‐abortion. We observed relatively similar patterns in both Nigeria and Côte d'Ivoire. Approximately one‐third of women in Nigeria (32.6 percent) and two‐fifths of women in Côte d'Ivoire (39.2 percent) were consistent contraceptive nonusers. Slightly more Ivorian women adopted contraception (28.5 percent) than Nigeria women (25.3 percent), but a higher percentage also discontinued contraception (12.6 percent in Côte d'Ivoire and 8.4 percent in Nigeria). Only one out of five (19.7 percent) Ivorian women continued use compared to one‐third of Nigerian women.

Table 2 shows the distribution of users and nonusers, with users further categorized by method effectiveness. Similar to the results from Figure 2, the proportion of users of effective and highly effective methods was higher post‐abortion compared to prior. In Nigeria, the use of effective short‐acting methods increased from 11.5 percent to 19.4 percent, and the use of highly effective methods increased from 2.7 percent to 8.7 percent; in Côte d'Ivoire, the use of effective methods increased from 13.3 percent to 31.7 percent, and the use of highly effective methods increased from 1.3 percent to 4.5 percent. However, in both countries, a higher proportion of pre‐abortion users shifted to less effective short‐acting or no method than shifted to more effective short‐acting or highly effective long‐acting methods post‐abortion. For example, in Nigeria, out of the 27.8 percent women using less effective short‐acting methods pre‐abortion, 6.2 percent stopped using afterward compared to only 4.8 percent (3.5 percent and 1.3 percent) who shifted to effective short‐acting or highly effective methods. Among the 11.5 percent of women who were using effective short‐acting methods pre‐abortion, 1.8 percent stopped using and 1.2 percent switched to a less effective short‐acting method compared to only 1.0 percent who switched to a highly effective long‐acting method. The same patterns were observed among women in Côte d'Ivoire; however, there was a decrease in the proportion of less effective short‐acting method users (from 17.8 percent pre‐abortion to 12.0 percent post‐abortion) because almost half (8.7 percent) stopped using after their abortion. Among adopters (women in the “no method” category before abortion but shifted to one of the three effectiveness categories after abortion), a higher proportion began using less effective short‐acting than effective or highly effective methods in Nigeria (12.8 percent vs. 8.1 percent and 4.5 percent, respectively), whereas in Côte d'Ivoire, adopters were more likely to use effective short‐acting methods versus less effective short‐acting or highly effective methods (19.4 percent vs. 6.5 percent and 2.6 percent).

**TABLE 2:** Distribution of contraceptive nonuse and method effectiveness before and after abortion, Nigeria and Côte d'Ivoire

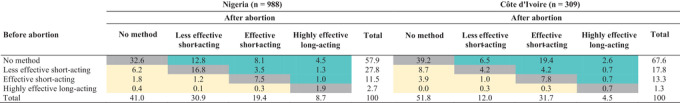	

NOTE: Proportions (%) account for clustering at the EA level. Highly effective long‐acting = sterilization, intrauterine device, and implant; effective short‐acting = pills, injectables, and diaphragm; and less effective short‐acting = condoms, other barrier methods, traditional methods, emergency contraception, and lactational amenorrhea method. Green cells refer to switching to more effective methods after abortion, whereas yellow cells refer to switching to less effective methods after abortion.

### Aim 2: Factors Associated with Contraceptive Adoption, Discontinuation, and Shifting to More Effective Methods Post‐Abortion

The results of the bivariate analysis for contraceptive adoption and discontinuation for both countries are presented in Appendix T1. In Côte d'Ivoire, higher proportions of women who wanted to talk to someone about contraception and who actually talked to someone about using contraception consistently used or adopted a method after the abortion. Women who attended secondary or higher education were more likely to consistently use or discontinue and less likely to consistently not use any method compared to those who never attended or attended primary school. In Nigeria, we observed generally higher levels of consistent use and adoption and lower levels of consistent nonuse and discontinuation among women who wanted to talk to someone about contraception, actually talked to someone about using contraception, and accessed a clinical source. Contraceptive dynamics patterns by education levels were less clear, similar to Côte d'Ivoire. More women who attended higher education levels were likely to consistently use a method compared to other education levels, whereas women who never attended school were more likely to consistently not use a method compared to other education levels. Higher levels of adoption were observed among women who attended only primary school.

Table 3 shows the results for the multivariable analysis in Nigeria only. Wanting to talk to someone about using contraception and accessing a clinical abortion source were associated with higher odds of contraceptive adoption (aOR: 1.66, 95 percent CI: 1.07–2.59; aOR: 1.67, 95 percent CI: 1.15–2.42, respectively) and decreased odds of contraceptive discontinuation (aOR: 0.47, 95 percent CI: 0.26–0.86; aOR: 0.54, 95 percent CI: 0.32–0.92, respectively). Women who actually talked to someone about contraception had higher odds of adoption only (aOR: 2.08, 95 percent CI: 1.36–3.17), while women who lived in urban areas had higher odds of discontinuation (aOR: 2.23, 95 percent CI: 1.12–4.46) compared to those from rural areas.

**TABLE 3:** Multivariable logistic regression examining factors associated with contraceptive adoption following abortion among pre‐abortion contraceptive nonusers, contraceptive discontinuation following abortion among pre‐abortion contraceptive users, and switching to a more effective method following abortion among continuing users, Nigeria

	Adoption among pre‐abortion nonusers		Discontinuation among pre‐abortion users		Switching to more effective method among continuing users	
	(n = 572)		(n = 416)		(n = 310)	
	aOR	95% CI	aOR	95% CI	aOR	95% CI
Age						
<20	1	(ref)	1	(ref)	1	(ref)
20–29	1.02	(0.65, 1.61)	0.90	(0.45, 1.80)	1.03	(0.46, 2.31)
30+	0.93	(0.54, 1.60)	0.70	(0.30, 1.67)	1.20	(0.44, 3.25)
Education						
Never	1	(ref)	1	(ref)	1	(ref)
Primary	2.24	(0.99, 5.07)	0.59	(0.19, 1.86)	**0.26**	**(0.07, 0.95)**
Secondary^a^	1.32	(0.67, 2.61)	0.41	(0.15, 1.12)	0.35	(0.12, 1.06)
Higher	1.91	(0.84, 4.31)	0.47	(0.16, 1.35)	‐	‐
Married (ref: not married)	1.32	(0.75, 2.32)	1.92	(0.77, 4.76)	1.04	(0.32, 3.32)
Wealth						
Lowest	1	(ref)	1	(ref)	1	(ref)
Middle	1.51	(0.85, 2.68)	0.98	(0.44, 2.21)	1.06	(0.30, 3.79)
Highest	1.59	(0.86, 2.95)	0.72	(0.30, 1.75)	2.10	(0.54, 8.19)
Residence (ref: rural)	0.91	(0.58, 1.42)	**2.23**	**(1.12, 4.46)**	0.69	(0.29, 1.65)
Had a child/children at time of abortion	0.80	(0.45, 1.43)	0.71	(0.27, 1.83)	2.03	(0.68, 6.11)
Wanted to talk to someone about contraception during abortion	**1.66**	**(1.07, 2.59)**	**0.47**	**(0.26, 0.86)**	1.37	(0.68, 2.76)
Talked to someone about using contraception during abortion	**2.08**	**(1.36, 3.17)**	0.86	(0.50, 1.48)	2.32	(1.00, 5.40)
Abortion source (ref: nonclinical)	**1.67**	**(1.15, 2.42)**	**0.54**	**(0.32, 0.92)**	1.21	(0.66, 2.24)
Felt she had a choice about using post‐abortion contraception	–	–	–	–	0.81	(0.12, 5.49)
Felt she had a choice about which method to use for post‐abortion contraception	–	–	–	–	2.58	(0.28, 24.12)

NOTE: Design‐based multivariable logistic regression adjusted for all covariates. Values in bold are p < 0.05.

^a^Secondary and higher levels of education combined for switching analysis.

aOR, adjusted odds ratio; CI, confidence interval; ref, reference.

Factors associated with shifts to more effective methods among continuing users in Nigeria are also presented in Table 3. Women who talked to someone about using contraception had borderline increased odds of adopting more effective methods after abortion (aOR: 2.32, 95 percent CI: 1.00–5.40), while women who attended primary education were less likely to adopt more effective methods compared to those who never went to school (aOR: 0.26, 95 percent CI: 0.07–0.95). Neither of the perceived contraceptive autonomy variables showed significant associations with shifting to more effective contraceptive methods.

### Aim 3: Correlates of Post‐Abortion Contraception

Bivariate analysis of contraceptive uptake after abortion found that in Nigeria, women who were using short‐acting or long‐acting methods pre‐abortion were more likely to use a method postabortion than women who were not using any method pre‐abortion (77.3 percent and 89.3 percent compared to 44.0 percent, respectively; results not shown in tables). Post‐abortion contraceptive use was higher among women who wanted to talk to someone about contraception compared to those who did not want to (74.0 percent vs. 53.7 percent), who talked to someone about contraception compared to those who did not (69.1 percent vs. 52.2 percent), and who accessed a clinical abortion source compared to those who accessed a nonclinical source (64.7 percent vs. 51.8 percent). Similarly, in Côte d'Ivoire, post‐abortion contraception was higher among women who wanted to talk to someone about contraception compared to those who did not want to (67.3 percent vs. 38.2 percent) and who talked to someone about contraception compared to those who did not (63.6 percent vs. 38.8 percent); however, unlike in Nigeria, pre‐abortion contraceptive use was only borderline significantly associated (p = 0.059) with post‐abortion use.

Table 4 shows the results from multivariable logistic regressions examining factors related to contraceptive use after an abortion in both countries. Contraceptive use before abortion was associated with increased odds of use after abortion in Nigeria only (aOR: 4.53, 95 percent CI: 3.22–6.37 for short‐acting methods; aOR: 5.65, 95 percent CI: 2.00–16.00 for long‐acting methods). Women who wanted to talk to someone about contraception (Nigeria aOR: 1.81, 95 percent CI: 1.27–2.56; Côte d'Ivoire aOR: 5.15, 95 percent CI: 2.64–10.06) and those who actually talked to someone about contraception (Nigeria aOR: 1.66, 95 percent CI: 1.18–2.33; Côte d'Ivoire aOR: 2.30, 95 percent CI: 1.23–4.30) were more likely to be using a method after their abortion in both countries. Accessing abortion via a clinical source was associated with greater odds of contraceptive uptake post‐abortion in Nigeria only (aOR: 1.70, 95 percent CI: 1.26–2.29), whereas having any children at the time of abortion was associated with increased odds of uptake in Côte d'Ivoire only (aOR: 2.12, 95 percent CI: 1.11–4.06).

**TABLE 4:** Multivariable logistic regression examining factors associated with contraceptive uptake after an abortion, Nigeria and Côte d'Ivoire

	Nigeria (n = 988)		Côte d'Ivoire (n = 309)	
	aOR	95% CI	aOR	95% CI
Age				
<20	1	(ref)	1	(ref)
20–29	1.07	(0.74, 1.57)	1.04	(0.55, 1.99)
30+	1.05	(0.66, 1.66)	0.78	(0.34, 1.78)
Education				
Never	1	(ref)	1	(ref)
Primary	2.01	(0.96, 4.2)	1.07	(0.50, 2.32)
Secondary^a^	1.58	(0.83, 2.98)	1.42	(0.58, 3.47)
Higher	1.94	(0.95, 3.97)	–	–
Married (ref: not married)	1.00	(0.61, 1.62)	0.64	(0.33, 1.22)
Wealth				
Lowest	1	(ref)	1	(ref)
Middle	1.33	(0.84, 2.11)	0.90	(0.42, 1.91)
Highest	1.47	(0.9, 2.42)	1.19	(0.51, 2.74)
Residence (ref: rural)	0.75	(0.51, 1.1)	0.76	(0.42, 1.40)
Had a child/children at time of abortion	0.98	(0.58, 1.65)	**2.12**	**(1.11, 4.06)**
Pre‐abortion contraception method use				
No method	1	(ref)	1	(ref)
Short‐acting method^b^	**4.53**	**(3.22, 6.37)**	1.30	(0.74, 2.28)
Long‐acting method	**5.65**	**(2.00, 16.00)**	–	–
Wanted to talk to someone about contraception during abortion	**1.81**	**(1.27, 2.56)**	**5.15**	**(2.64, 10.06)**
Talked to someone about using contraception during abortion	**1.66**	**(1.18, 2.33)**	**2.30**	**(1.23, 4.30)**
Source of last method (ref: nonclinical)	**1.70**	**(1.26, 2.29)**	0.85	(0.48, 1.50)

NOTE: Design‐based multivariable logistic regression adjusted for all covariates. Values in bold are p < 0.05.

^a^ Secondary and higher levels of education combined for Côte d'Ivoire.

^b^ Short‐acting and long‐acting methods combined for Côte d'Ivoire.

aOR, adjusted odds ratio; CI, confidence interval; ref, reference.

We conducted additional analyses in Nigeria to examine the extent to which women received contraceptive counseling and were therefore more likely to talk to someone about contraception when accessing a clinical abortion source. Although we did not find a difference in the proportion of women who talked to someone about contraception between those who accessed their abortion from a clinical source (39.0 percent, 95 percent CI: 33.5–44.8) versus a nonclinical source (36.6 percent, 95 percent CI: 31.0‐42.6), a majority of women (80.9 percent, 95 percent CI: 73.3–86.8) who accessed a clinical source and talked to someone about contraception used a method after the abortion compared to only 55.0 percent (95 percent CI: 45.4–64.3) of women who accessed a clinical source but did not talk to someone about contraception (estimates not shown in tables).

## DISCUSSION

Our results are consistent with prior studies examining contraceptive dynamics, demonstrating improved contraceptive use after an abortion in two West African countries where abortion is highly legally restricted, and contribute new findings with regard to factors related to post‐abortion contraceptive use, particularly contraceptive communication and access to a clinical source. While most women did not change their contraceptive status after their abortion, those who did were more likely to adopt a method than discontinue one. The use of long‐acting methods saw a modest increase after an abortion; however, short‐acting methods remained the much more popular choice among contraceptive users. The increase in contraceptive use effectiveness after an abortion was mostly driven by pre‐abortion nonusers adopting a method rather than pre‐abortion users shifting to more effective methods. We also observed a difference in contraceptive uptake among adopters in both countries. In Nigeria, adopters were more likely to use less effective short‐acting methods (condoms, other barrier methods, traditional methods, emergency contraception, and lactational amenorrhea method), whereas adopters in Côte d'Ivoire were more likely to choose effective short‐acting methods (pills, injectables, and diaphragms). This difference is in line with the method preferences in each country: injectables and pills are more common in Côte d'Ivoire, while condoms and traditional methods are more common in Nigeria (PMA 2018, 2021).

In both settings, a desire to talk about contraceptive methods and actually talking to someone about contraceptive methods was significantly associated with contraceptive use, which provides new insight into factors influencing post‐abortion contraceptive use dynamics. In Nigeria, wanting to have conversations about contraception during abortion care was associated with both increased adoption among pre‐abortion nonusers and decreased discontinuation among pre‐abortion users. In addition, talking to someone about using contraception was associated with higher adoption among pre‐abortion nonusers. Both factors were associated with post‐abortion contraceptive use in Nigeria and Côte d'Ivoire. The desire to discuss contraception with someone may be due to women being more motivated to use any or a different contraceptive method after terminating a pregnancy to prevent future unintended pregnancies and therefore seeking information about their options (Penfold et al. 2018). Alternatively, wanting to talk to someone about contraception may precede the development of the intent to use contraception after the abortion, in which case they may be using the information obtained from the discussion to inform whether they intend to use a contraceptive method. Regardless of the mechanism, when women talk to someone about contraception during their abortion, it is an opportunity for them to receive information that helps them make informed decisions that meet their reproductive needs. A qualitative study in Nepal, for example, found that women who received contraceptive information during their abortion care felt more empowered to make informed decisions and reported that it eased their concerns regarding negative side effects (Rogers et al. 2019). It is critical to offer women quality, voluntary post‐abortion contraceptive information and ensure that these services are accessible to those who self‐manage their abortions outside the formal health care system. However, access to contraceptive information does not guarantee contraceptive uptake. Some women choose to delay or forgo the decision completely because of previous negative experiences with methods, not having found a method that fits their needs, or feeling that the information they received during their care was inadequate (Rogers et al. 2019; Penfold et al. 2018; Mutua et al. 2017).

Access to a clinical abortion source was associated with higher adoption and lower discontinuation compared to accessing a nonclinical abortion source among Nigerian women. In addition, our examination of post‐abortion contraceptive use found that abortion source was associated with improved uptake in Nigeria. Other research from this study found that three out of five women in Nigeria (62.2 percent) and Côte d'Ivoire (59.5 percent) access care outside of the formal health care setting (Bell et al. 2019), precluding women from receiving post‐abortion contraceptive counseling unless they experience abortion complications that necessitate accessing clinical care. In sub‐Saharan Africa, studies have shown that women are more likely to use contraception after receiving PAC services (Hagos et al. 2018; Tripney et al. 2013; Johnson et al. 2002); however, the quality of services varies. A study in Kenya found that a higher proportion of public facilities offered PAC services on discharge compared to private facilities (Mutua et al. 2017). A qualitative study in Nepal found that women who accessed clinical abortion sources reported receiving family planning counseling, whereas those who accessed pharmacies did not (Rogers et al. 2019). While expanding the legal indications for safe abortion in these settings would help to increase the likelihood that women access facility care and receive PAC or practice self‐care that is integrated within the formal health care system, women are not guaranteed to receive contraceptive counseling in a clinical setting due to existing gaps in the quality of services. Examination of post‐abortion contraceptive use in Nigeria in this study revealed variations in PAC quality, as more than half of women who received care from a clinical source reported not discussing contraception during their abortion experience. It is thus essential to improve PAC services so women receive comprehensive, high‐quality contraceptive counseling to enable them to make an informed choice that meets their fertility needs.

Although this study confirms that PAC presents an opportunity for women to learn about family planning and the different methods available to them, some results in our analyses require further investigation. We found increased adoption among women who never went to school compared to those who attended primary education. This finding could be due to provider bias based on client characteristics such as education, age, and parity that may influence contraceptive counseling, decision‐making, and provider–patient interactions (Dehlendorf et al. 2010; Gilliam 2015). We also need to examine reasons why some women who talk about contraception during their abortion do not end up using a method to better understand whether this is due to personal preference or further barriers to access care.

A strength of this study is its use of data from population‐based samples of reproductive age women in Nigeria and Côte d'Ivoire who reported an abortion. Our sample included women who terminated pregnancies entirely outside of the formal health care system, which is a population often omitted from existing literature on post‐abortion contraceptive uptake. We also had rich data on women's background characteristics, fertility intentions, and abortion care experience—variables that are not captured in facility data that many previous studies have used. Additionally, we were able to examine contraceptive dynamics surrounding women's abortion in two West African settings, increasing the robustness and generalizability of our findings.

However, this study is not without limitations. First, small sample sizes for both countries limited the power of our multivariable regression models examining contraceptive dynamics. Although we were able to assess adoption and discontinuation in Nigeria, the effectiveness analysis was limited to women who shifted to more effective methods but not those who shifted to less effective methods because of the small sample of eligible women. For Côte d'Ivoire, our analyses were even more limited due to the much smaller sample. Second, there is the potential for bias from underreporting of abortion in the baseline survey. That is, the results could be biased if the likelihood of using contraception before or after an abortion is associated with the likelihood of reporting an abortion and other sociodemographic or abortion characteristics. This differential reporting is particularly problematic for sensitive self‐reported sexual behaviors, such as abortion, which this study relies on. Women may overreport their contraceptive use because of their perceived social acceptability or to frame the unintended pregnancy in terms of contraceptive failure (Trussell and Vaughan 1999), whereas abortion may be underreported because it is illegal or socially stigmatized (Stuart and Grimes 2009). Another limitation is the potential for recall bias in self‐reporting of pre‐abortion and post‐abortion contraceptive use or changes in the service delivery environment around an event taking place several years prior to the follow‐up survey that could change observed relationships. However, this does not seem to be a substantial concern for our study; when we examined abortions in the last five years compared to abortions from more than five years prior to the baseline survey, the odds ratios were in the same direction for the variables that were significant in our main model, indicating that these relationships were fairly stable over time. Finally, while our data enabled us to explore covariates that were often not collected in previous studies, further details into women's abortion experience may help shed more light on the significant associations we observed, specifically the content and dynamics of conversations about contraception, partner support, costs, preferences, and quality of PAC services.

### Implications for Practice

Our results suggest that communicating about contraception after an abortion is a key factor in influencing post‐abortion contraceptive uptake. Efforts to improve the availability and quality of PAC contraceptive counseling are needed to ensure that women who access abortion services in clinical settings receive quality contraceptive counseling and services from skilled providers as part of PAC. However, these improvements will not necessarily reach women who rely on medication abortion (or nonrecommended methods) from nonclinical sources. As women in sub‐Saharan Africa and elsewhere increasingly rely on self‐managed medication abortion to terminate a pregnancy outside the formal health care system, we need to consider how to effectively meet their PAC contraceptive needs. Although PAC will always require a clinical component for the treatment of complications, the increasing use of self‐managed medication abortion may require a shift in the provision of PAC contraceptive counseling beyond the clinical abortion experience. Governments and implementing partners, for example, can expand existing family planning counseling training to vendors and community pharmacists outside the formal health care system to include instructions on referrals to health facilities for abortion clients to receive comprehensive PAC services. Such strategies that leverage interactions with nonclinical abortion sources to try to link women back to the formal health sector for contraceptive counseling post‐abortion may be needed to ensure that women have access to information that can help them fulfill their reproductive goals.

## CONCLUSION

Abortion care presents an important window of opportunity to improve contraceptive uptake and adoption of effective and highly effective methods among women in Nigeria and Côte d'Ivoire. Adoption and continuation were higher among those who discussed contraception and received care from a clinical source, highlighting the importance of comprehensive abortion care, including post‐abortion contraceptive counseling. Among women who rely on nonclinical sources of care, it is still critical to connect them to PAC in the formal health system so they can receive high‐quality contraceptive counseling to make informed decisions that are aligned with their reproductive needs and goals.

## CONFLICTS OF INTEREST

The authors have no conflicts of interest to disclose.

## ETHICS APPROVAL AND PATIENT CONSENT STATEMENT

The study protocol was approved by the Johns Hopkins Bloomberg School of Public Health Institutional Review Board, the National Health Research Ethics Committee of Nigeria, and the Comité National d’Éthique des Sciences de la Vie et de la Santé (CNESVS) of the Côte d'Ivoire Ministry of Health and Public Hygiene. All respondents gave their consent to take part in the study.

## Supporting information

APPENDIX T1 Respondent and abortion‐related characteristics by contraceptive behavior before and after an abortion, Nigeria and Côte d'Ivoire

## Data Availability

Data for this study will be publicly available at pmadata.org. Once posted online, anyone can access after completing a brief request form at https://www.pmadata.org/data/available‐datasets.
