# Supplementary material for: Contraceptive Use Before and After Abortion: A Cross‐Sectional Study from Nigeria and Côte d'Ivoire
Source: Stud Fam Plann. 2022 Jul 20;53(3):433–53. doi: 10.1111/sifp.12208 (PMC9545736; doi:10.1111/sifp.12208)
Supplement: Supplementary file 1 — APPENDIX T1 Respondent and abortion‐related characteristics by contraceptive behavior before and after an abortion, Nigeria and Côte d'Ivoire [file SIFP-53-433-s001.docx]

**APPENDIX**

**T1 Respondent and abortion-related characteristics by contraceptive behavior before and after an abortion, Nigeria and Côte d’Ivoire**

|  | **Nigeria (n = 988)** | | | | | | | | | | **Côte d'Ivoire (n = 309)** | | | | | | | | | |
| --- | --- | --- | --- | --- | --- | --- | --- | --- | --- | --- | --- | --- | --- | --- | --- | --- | --- | --- | --- | --- |
|  | **Consistent non-use** | | **Consistent use** | | **Discontinued** | | **Adopted** | | **Total n** | **Chi-square** | **Consistent non-use** | | **Consistent use** | | **Discontinued** | | **Adopted** | | **Total n** | **Chi-square** |
|  | n | % | n | % | n | % | n | % |  |  | n | % | n | % | n | % | n | % |  |  |
| Total | 322 | 32.6 | 333 | 33.7 | 83 | 8.4 | 250 | 25.3 | 988 |  | 121 | 39.2 | 61 | 19.7 | 39 | 12.6 | 88 | 28.5 | 309 |  |
| Age |  |  |  |  |  |  |  |  |  | 0.693 |  |  |  |  |  |  |  |  |  | 0.396 |
| <20 | 79 | 36.1 | 68 | 31.1 | 17 | 7.8 | 55 | 25.1 | 219 |  | 52 | 38.0 | 31 | 22.6 | 20 | 14.6 | 34 | 24.8 | 137 |  |
| 20-29 | 140 | 29.5 | 168 | 35.4 | 44 | 9.3 | 122 | 25.7 | 474 |  | 48 | 39.7 | 24 | 19.8 | 13 | 10.7 | 36 | 29.8 | 121 |  |
| 30+ | 94 | 33.3 | 94 | 33.3 | 22 | 7.8 | 72 | 25.5 | 282 |  | 19 | 41.3 | 5 | 10.9 | 6 | 13.0 | 16 | 34.8 | 46 |  |
| Education |  |  |  |  |  |  |  |  |  | **<0.001** |  |  |  |  |  |  |  |  |  | **0.037** |
| Never | 43 | 45.3 | 23 | 24.2 | 10 | 10.5 | 19 | 20.0 | 95 |  | 45 | 45.5 | 13 | 13.1 | 11 | 11.1 | 30 | 30.3 | 99 |  |
| Primary | 36 | 31.0 | 32 | 27.6 | 9 | 7.8 | 39 | 33.6 | 116 |  | 54 | 45.4 | 22 | 18.5 | 13 | 10.9 | 30 | 25.2 | 119 |  |
| Secondary* | 182 | 36.0 | 160 | 31.7 | 35 | 6.9 | 128 | 25.3 | 505 |  | 22 | 24.2 | 26 | 28.6 | 15 | 16.5 | 28 | 30.8 | 91 |  |
| Higher | 60 | 22.1 | 118 | 43.5 | 29 | 10.7 | 64 | 23.6 | 271 |  |  | | | | | | | | |  |
| Married |  |  |  |  |  |  |  |  |  | 0.161 |  |  |  |  |  |  |  |  |  | 0.589 |
| No | 154 | 35.8 | 136 | 31.6 | 30 | 7.0 | 110 | 25.6 | 430 |  | 63 | 36.4 | 38 | 22.0 | 23 | 13.3 | 49 | 28.3 | 173 |  |
| Yes | 167 | 30.0 | 197 | 35.4 | 53 | 9.5 | 140 | 25.1 | 557 |  | 58 | 42.6 | 23 | 16.9 | 16 | 11.8 | 39 | 28.7 | 136 |  |
| Wealth |  |  |  |  |  |  |  |  |  | 0.109 |  |  |  |  |  |  |  |  |  | 0.108 |
| Lowest | 80 | 40.0 | 63 | 31.5 | 16 | 8.0 | 41 | 20.5 | 200 |  | 42 | 44.2 | 16 | 16.8 | 9 | 9.5 | 28 | 29.5 | 95 |  |
| Middle | 119 | 33.7 | 107 | 30.3 | 29 | 8.2 | 98 | 27.8 | 353 |  | 51 | 44.7 | 21 | 18.4 | 13 | 11.4 | 29 | 25.4 | 114 |  |
| Highest | 122 | 28.2 | 161 | 37.3 | 38 | 8.8 | 111 | 25.7 | 432 |  | 27 | 27.3 | 24 | 24.2 | 17 | 17.2 | 31 | 31.3 | 99 |  |
| Residence (urban) |  |  |  |  |  |  |  |  |  | 0.058 |  |  |  |  |  |  |  |  |  | 0.413 |
| No | 136 | 37.1 | 122 | 33.2 | 22 | 6.0 | 87 | 23.7 | 367 |  | 49 | 41.2 | 17 | 14.3 | 17 | 14.3 | 36 | 30.3 | 119 |  |
| Yes | 186 | 30.0 | 211 | 34.0 | 61 | 9.8 | 163 | 26.2 | 621 |  | 72 | 37.9 | 44 | 23.2 | 22 | 11.6 | 52 | 27.4 | 190 |  |
| Had a child/children at time of abortion |  |  |  |  |  |  |  |  |  | 0.580 |  |  |  |  |  |  |  |  |  | 0.586 |
| No | 157 | 34.7 | 145 | 32.1 | 36 | 8.0 | 114 | 25.2 | 452 |  | 54 | 41.2 | 25 | 19.1 | 19 | 14.5 | 33 | 25.2 | 131 |  |
| Yes | 165 | 30.8 | 188 | 35.1 | 47 | 8.8 | 136 | 25.4 | 536 |  | 67 | 37.6 | 36 | 20.2 | 20 | 11.2 | 55 | 30.9 | 178 |  |
| Wanted to talk to someone about contraception during abortion |  |  |  |  |  |  |  |  |  | **<0.001** |  |  |  |  |  |  |  |  |  | **<0.001** |
| No | 258 | 37.6 | 200 | 29.1 | 62 | 9.0 | 167 | 24.3 | 687 |  | 102 | 56.0 | 17 | 9.3 | 24 | 13.2 | 39 | 21.4 | 182 |  |
| Yes | 58 | 19.8 | 132 | 45.1 | 21 | 7.2 | 82 | 28.0 | 293 |  | 17 | 13.8 | 44 | 35.8 | 14 | 11.4 | 48 | 39.0 | 123 |  |
| Talked to someone about using contraception during abortion |  |  |  |  |  |  |  |  |  | **<0.001** |  |  |  |  |  |  |  |  |  | **<0.001** |
| No | 226 | 39.8 | 166 | 29.2 | 47 | 8.3 | 129 | 22.7 | 568 |  | 87 | 52.4 | 25 | 15.1 | 21 | 12.7 | 33 | 19.9 | 166 |  |
| Yes | 93 | 22.5 | 166 | 40.1 | 36 | 8.7 | 119 | 28.7 | 414 |  | 34 | 23.8 | 36 | 25.2 | 18 | 12.6 | 55 | 38.5 | 143 |  |
| Source of last abortion method |  |  |  |  |  |  |  |  |  | **0.002** |  |  |  |  |  |  |  |  |  | 0.577 |
| Non-clinical | 181 | 36.1 | 163 | 32.5 | 52 | 10.4 | 106 | 21.1 | 502 |  | 91 | 41.6 | 43 | 19.6 | 26 | 11.9 | 59 | 26.9 | 219 |  |
| Clinical | 141 | 29.0 | 170 | 35.0 | 31 | 6.4 | 144 | 29.6 | 486 |  | 30 | 33.3 | 18 | 20.0 | 13 | 14.4 | 29 | 32.2 | 90 |  |
| Used Rao-Scott corrected chi-square statistic. Proportions (%) account for clustering at the EA level. *Secondary and higher education were combined for Côte d’Ivoire. | | | | | | | | | | | | | | | | | | | | |
